# Diagnostic Dilemmas in Cardiac Transthyretin Amyloidosis With Coexistent Low-Grade B-Cell Lymphoma: A Case Report

**DOI:** 10.7759/cureus.91227

**Published:** 2025-08-29

**Authors:** Ojas Bansal, Rohini Garg, Hemani Raj Sharma, Laila A Payvandi

**Affiliations:** 1 Cardiology, Banner Health, Mesa, USA; 2 Hospital Medicine, Mercy Hospital Council Bluffs, Council Bluffs, USA; 3 Bachelors of Science, UC Berkeley, Berkeley, USA; 4 Cardiology, UnityPoint Health, Cedar Rapids, USA

**Keywords:** congestive heart failure (chf), endomyocardial biopsy, large b cell lymphomas, tc-99m pyp imaging, ttr cardiac amyloidosis

## Abstract

Cardiac amyloidosis, though a rare clinical entity, is increasingly recognized as an important etiology of heart failure and cardiac arrhythmias. The deposition of misfolded amyloid fibrils in the myocardium leads to restrictive cardiomyopathy and progressive cardiac dysfunction. Two main precursor proteins are implicated in cardiac amyloidosis: transthyretin (TTR), synthesized by the liver, and immunoglobulin light chain (AL), usually associated with plasma cell dyscrasias and lymphomas. Given the substantial differences in treatment and prognosis between TTR and AL amyloidosis, accurate differentiation between these subtypes is critical for delivering optimal patient care.

We report the case of a 64-year-old man with progressive dyspnea and atrial flutter who was found to have severe concentric left ventricular hypertrophy and a characteristic “cherry on top” strain pattern on echocardiography. A technetium-99m pyrophosphate (PYP) scan demonstrated grade 3 uptake, but a concurrent abnormal IgM spike on serum electrophoresis prompted further hematologic evaluation. Bone marrow biopsy revealed a low-grade B-cell lymphoma without amyloid deposition, while endomyocardial biopsy with mass spectrometry confirmed wild-type TTR amyloidosis. The patient underwent atrial flutter ablation and was initiated on tafamidis with clinical improvement. To our knowledge, this represents the first reported case of coexisting ATTR cardiac amyloidosis and B-cell lymphoma diagnosed simultaneously. This case highlights the diagnostic challenges when hematologic malignancy and cardiac amyloidosis coexist, underscores the importance of endomyocardial biopsy for definitive typing, and emphasizes the role of a multidisciplinary approach in guiding management and optimizing outcomes.

## Introduction

Cardiac amyloidosis (CA) is caused by the deposition of insoluble misfolded precursor amyloid proteins in the cardiac extracellular space. More than 36 different amyloid precursor proteins are associated with systemic amyloidosis [1]. However, cardiac amyloidosis is predominantly linked to two main precursor proteins: transthyretin (TTR) and immunoglobulin light chain (AL) [2]. TTR is a tetrameric protein synthesized by the liver that normally transports thyroid hormone and retinol. TTR amyloidosis is subdivided into two main categories: hereditary amyloidosis (h-ATTR), caused by genetic mutations in the transthyretin gene, and wild-type amyloidosis (w-ATTR), caused by the deposition of wild-type (normal) transthyretin, typically seen in older patients.

AL amyloidosis results from the deposition of proteins derived from immunoglobulin light chain fragments. This usually occurs as a complication of plasma cell dyscrasias such as multiple myeloma, Waldenström macroglobulinemia, or, less commonly, non-Hodgkin lymphoma or chronic lymphocytic lymphoma [3].

Cardiac amyloidosis has a pathognomonic appearance on transthoracic echocardiogram (TTE) and cardiac MRI. Further diagnostic workup is needed to differentiate between AL and TTR amyloidosis. In the absence of monoclonal proteins, a diagnosis of TTR cardiac amyloidosis can be made noninvasively with technetium-99m pyrophosphate scintigraphy (PYP). However, the presence of abnormal monoclonal proteins poses a diagnostic dilemma and warrants further evaluation with tissue biopsy and mass spectrometry analysis to differentiate between AL and TTR cardiac amyloidosis [4].

Clinically, the coexistence of B-cell lymphoma and cardiac amyloidosis (ATTR-CA) is typically associated with AL amyloidosis, which requires urgent chemotherapy directed at the underlying lymphoma due to its aggressive course. In contrast, when cardiac involvement is due to TTR amyloidosis, as in this case, treatment is directed toward the amyloid itself, while the malignancy may not require immediate therapy. This distinction has critical implications for patient management and prognosis.

This article was previously presented as an oral presentation at the 2024 ASNC Annual Scientific Session and Exhibition on September 5, 2024.

## Case presentation

A 64-year-old Caucasian man presented to the office with a six-month history of shortness of breath consistent with NYHA class II symptoms. At baseline, he was able to perform more than 10 METS, and it was unusual for him to experience dyspnea with exertion. He was referred to our center for further evaluation and management. He was a nonsmoker with no prior cardiac history.

On examination, he appeared comfortable. His lungs were clear to auscultation without rales or rhonchi. Cardiac examination showed an irregular heart rhythm with no murmurs and no jugular venous distension. Peripheral pulses were equal, and there was no lower extremity edema.

**Figure 1:**
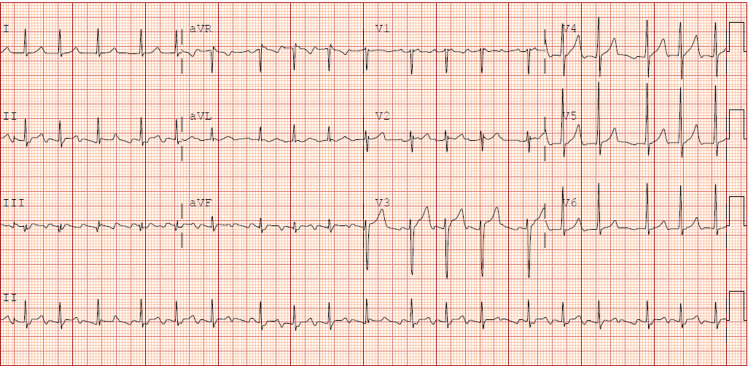
EKG showing atrial flutter with controlled ventricular rate

His initial evaluation in the office with an EKG showed atrial flutter with a controlled heart rate of 62 bpm, without any AV nodal blocker medications (see Figure 1).

**Figure 2:**
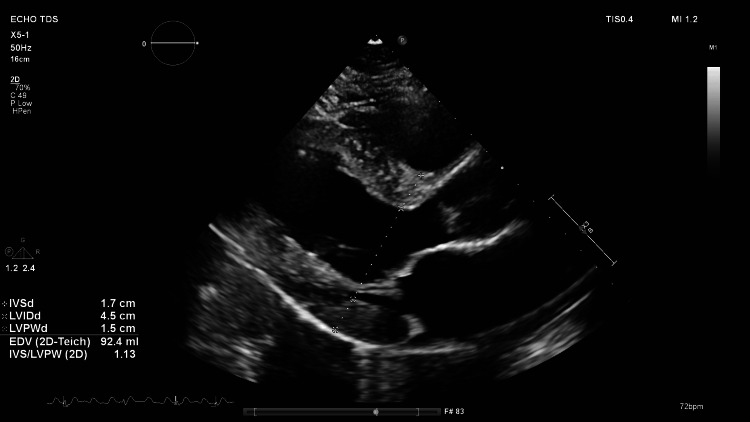
Echocardiogram showing severe left ventricular hypertrophy with bright and echogenic endocardium from amyloid infiltration

**Figure 3:**
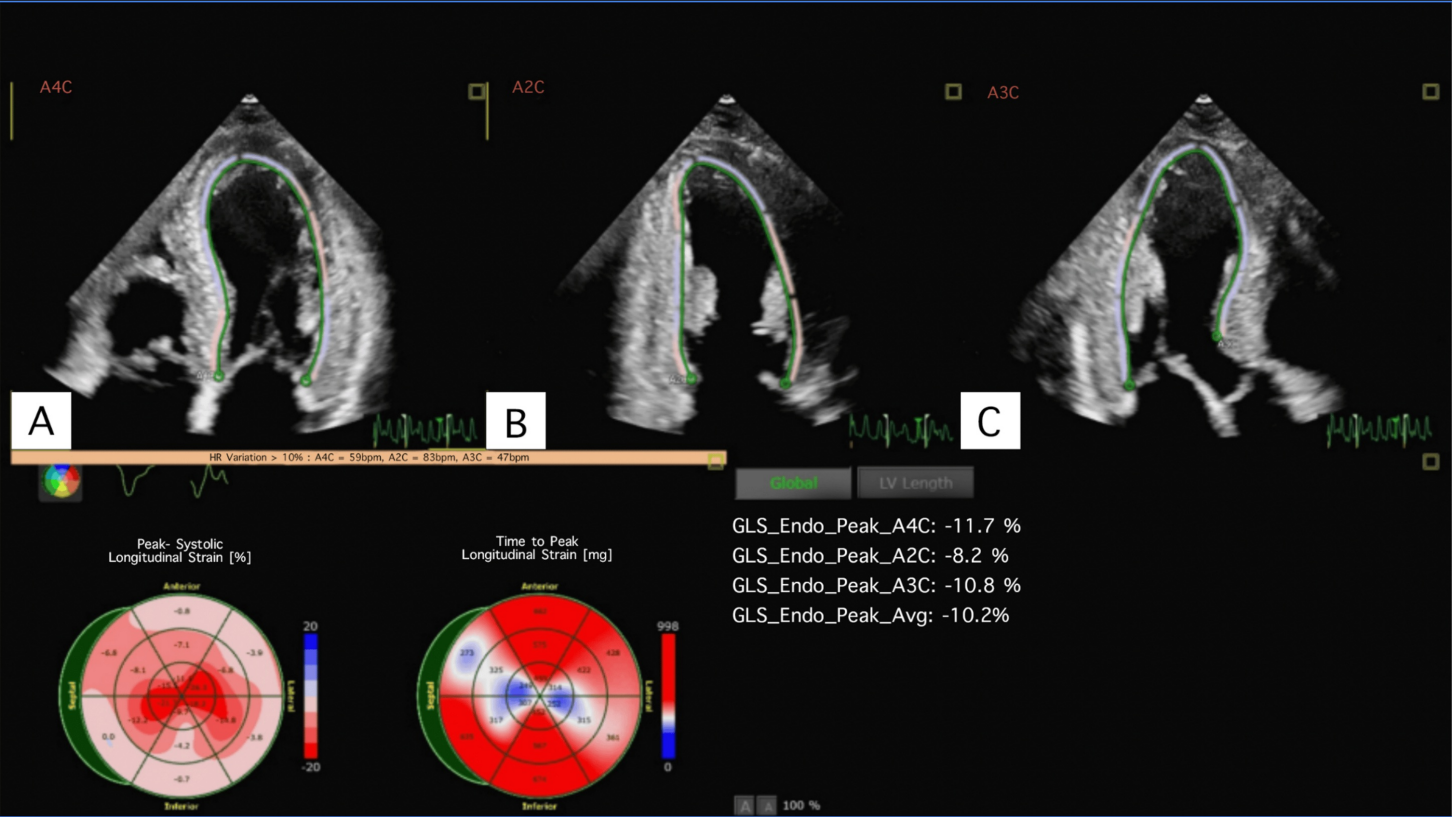
Classic strain pattern demonstrated in panel A (apical 4-chamber), panel B (apical 2-chamber), and panel C (apical 3-chamber). The pattern, associated with cardiac amyloidosis, shows reduced overall global longitudinal strain with relative apical sparing, referred to as the “cherry on top” sign

His echocardiogram showed moderate to severe concentric hypertrophy of the left ventricular myocardium with thick, echogenic endocardium suspicious for infiltrative cardiomyopathy (see Figure 2). His LVEF was normal at 62%. The left atrium was severely dilated without any significant valvular abnormalities or pericardial effusion. Strain imaging showed evidence of reduced global longitudinal strain (GLS) of -10% with relative sparing of the left ventricular apex (see Figure 3). A markedly reduced GLS value, such as -10%, serves as an independent predictor of adverse outcomes in patients with cardiac amyloidosis. It reflects significant impairment in the myocardium’s contractile function, primarily due to infiltration of amyloid proteins.

**Figure 4:**
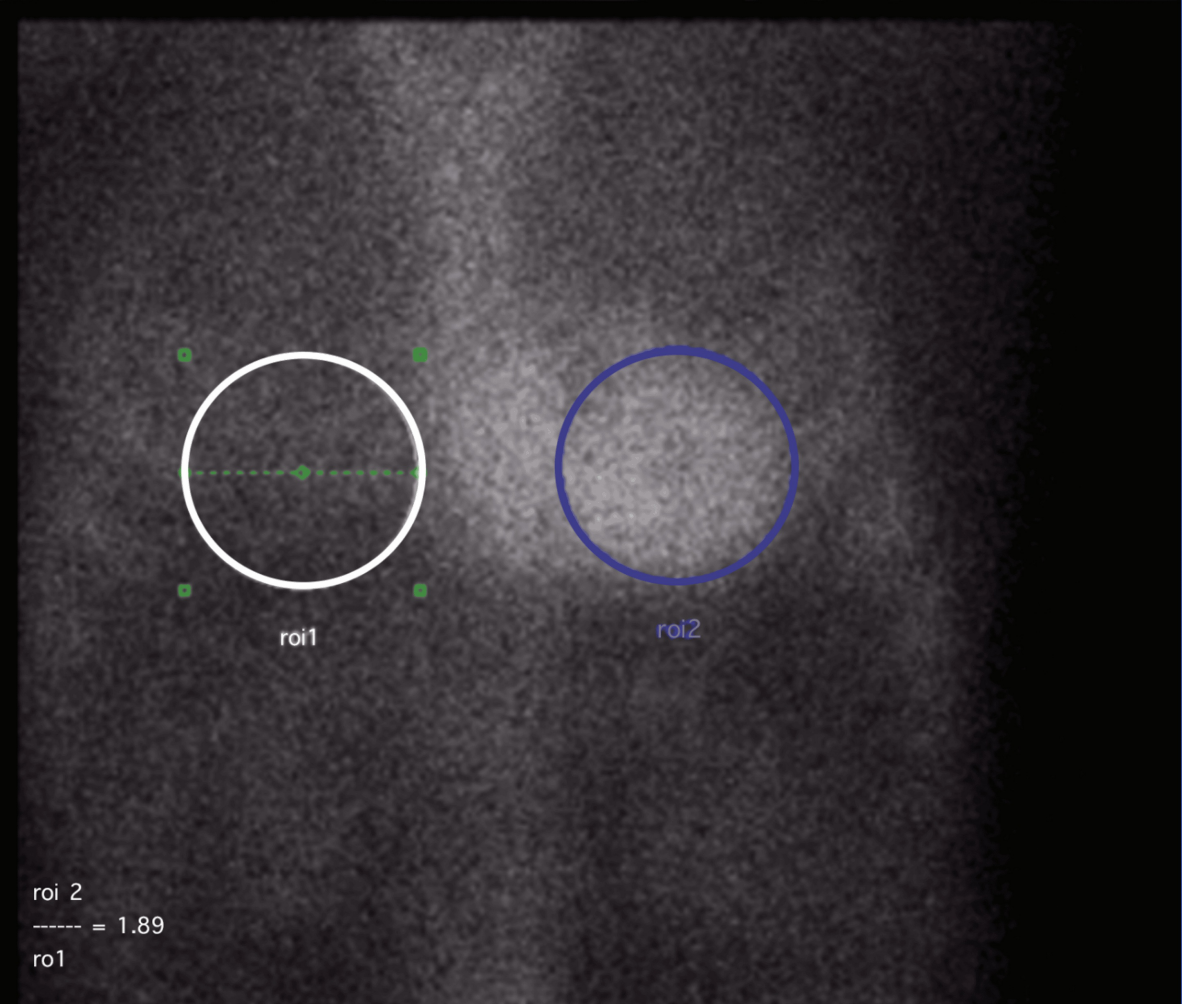
Technetium-99m pyrophosphate (PYP) scintigraphy showing strong positivity with grade 3 uptake on the Perugini scale and a heart-to-contralateral (H/CL) ratio of 1.9

He underwent further workup with serum and urine protein electrophoresis and kappa/lambda light chain assays. Serum electrophoresis was abnormal, showing an IgM spike. In the interim, he had a technetium-99m PYP scintigraphy, which was strongly positive with grade 3 uptake on the Perugini scale and an HCL ratio of 1.89 (see Figure 4).

The abnormal serum electrophoresis raised concern for a false-positive PYP scan, and he was referred to hematology for further evaluation. A bone marrow biopsy revealed a low-grade B-cell lymphoproliferative disorder involving approximately 5-10% of the marrow.

He subsequently underwent an endomyocardial biopsy that confirmed ATTR (transthyretin)-type cardiac amyloidosis. Mass spectrometry was most consistent with age-related (wild-type) cardiac amyloidosis. He underwent atrial flutter ablation and was able to maintain sinus rhythm. Pharmacotherapy with tafamidis, an amyloid fibril stabilizer, was initiated. At his most recent six-month follow-up, his LVEF remained stable with no further disease progression, and his dyspnea improved to NYHA class I.

## Discussion

In this case, we demonstrate a patient with a concurrent diagnosis of TTR cardiac amyloidosis and B-cell lymphoma that was unrelated to the cardiac amyloid. To our knowledge, this is the first reported case of these two distinct clinical entities. We will discuss the pathophysiology of the two main types of cardiac amyloidosis and the diagnostic workup involved in such challenging cases.

Pathophysiology of amyloidosis

Amyloid protein is an amorphous fibrillar material that can deposit in multiple organ systems. There are over 36 different amyloid precursor proteins associated with systemic amyloidosis. However, cardiac amyloidosis is predominantly associated with two main precursor proteins: immunoglobulin light chain (AL) and transthyretin (TTR) amyloid [2].

AL amyloidosis is due to the deposition of protein derived from immunoglobulin light chain fragments. It is a multi-organ disease, with cardiac involvement being the second most commonly affected system. Most cases of AL amyloidosis are associated with plasma cell neoplasms. AL amyloidosis associated with B-cell non-Hodgkin lymphoma is rare and is usually IgM-related. About 5-7% of cases of AL amyloidosis are associated with IgM paraprotein [3]. In a study of 250 patients with systemic AL amyloidosis associated with IgM, about 54% were found to be related to non-Hodgkin lymphoma (NHL) as the underlying disorder [4]. Notably, cardiac involvement is less common in IgM-related AL amyloidosis compared to non-IgM-related AL amyloidosis [4]. Median survival with AL cardiac amyloidosis is approximately 1-3 months and warrants urgent treatment [5]. Therapy focuses on treating the underlying lymphoma, and autologous stem cell transplantation may be considered in select cases [3].

TTR is a tetrameric protein synthesized by the liver that normally transports thyroid hormone and retinol. TTR amyloidosis is subdivided into two main categories: hereditary amyloidosis (h-ATTR), caused by mutations in the transthyretin gene, and wild-type amyloidosis (w-ATTR), caused by deposition of normal transthyretin, typically seen in older patients. Wild-type TTR predominantly causes cardiomyopathy, though it may also manifest as carpal tunnel syndrome. h-ATTR is inherited in an autosomal dominant fashion and may involve both the heart and peripheral nervous system [6,7].

The precise identification of the precursor protein is crucial because different forms of amyloidosis have distinct clinical courses and require entirely different therapeutic approaches, ranging from symptomatic management to targeted therapies aimed at preventing or reducing amyloid deposition.

Diagnosis of different types of cardiac amyloidosis

Cardiac amyloidosis is initially suspected on transthoracic echocardiography. Amyloid deposition in the heart can lead to increased thickness of the ventricular walls, particularly the left ventricle. This is often observed as an increase in the echogenicity (brightness) of the myocardium. Additional findings include biatrial enlargement, grade 2 or higher diastolic dysfunction, and pericardial effusion. Strain imaging aids in the diagnosis, with a classic pattern of reduced global longitudinal strain and relative apical sparing, often referred to as the “cherry on top” sign (as noted in Figure 3). The next step is serum and urine protein electrophoresis. Multidisciplinary collaboration is needed to accurately interpret monoclonal light chain testing results, to carry out and interpret cardiac scintigraphy, and to determine whether a tissue biopsy is warranted.

In patients with normal serum protein electrophoresis, a PYP cardiac scintigraphy scan should be performed, as it has a 99% positive predictive value for TTR amyloidosis.

If monoclonal protein is detected on serum or urine immunofixation electrophoresis or if the serum free light chain ratio is abnormal, histological confirmation with tissue biopsy is required. Indications for endomyocardial biopsy in suspected cardiac amyloidosis include monoclonal protein on serum or urine immunofixation electrophoresis or an abnormal serum free light chain ratio, a negative or equivocal Tc-PYP scan in the setting of high clinical suspicion, or lack of availability of cardiac scintigraphy [8].

Our patient had no prior known hematological disorder, and baseline laboratory work was unremarkable. He underwent a PYP scan for further evaluation, which came back strongly positive. But in our case, the IgM protein was abnormal, and the patient was referred to a hematologist for a bone marrow biopsy. He was diagnosed with B-cell lymphoma with 5-10% involvement of the bone marrow. The bone marrow biopsy did not show any evidence of amyloid deposition. He was then referred for an endomyocardial biopsy that showed ATTR (transthyretin)-type cardiac amyloidosis. Mass spectrometry results were most consistent with age-related (wild-type) cardiac amyloidosis.

## Conclusions

To our knowledge, this is the first reported case of ATTR cardiac amyloidosis in a patient with a co-existing B-cell lymphoma, and neither diagnosis was previously known. Our case underscores the importance of discerning the exact type of amyloid in a case of cardiac amyloidosis when the technetium-99m PYP scintigraphy is positive, but serum or urine electrophoresis is abnormal. The gold standard for diagnosing and typing cardiac amyloidosis is an endomyocardial biopsy. A multidisciplinary approach, involving specialists from cardiology, hematology, and oncology, is essential for accurate diagnosis and optimal treatment outcomes. Early and accurate diagnosis is important for early intervention, as the treatment varies depending on the type of amyloidosis. With the availability of medications that have been shown to halt the disease progression in ATTR amyloidosis, it is paramount that we aim for early and accurate diagnosis, so we can offer appropriate treatment and potentially improve survival. This nuanced approach highlights the need for individualized treatment strategies guided by a comprehensive understanding of disease dynamics.
